# Delta-radiomics models based on multi-phase contrast-enhanced magnetic resonance imaging can preoperatively predict glypican-3-positive hepatocellular carcinoma

**DOI:** 10.3389/fphys.2023.1138239

**Published:** 2023-08-03

**Authors:** Zewen Han, Hanting Dai, Xiaolin Chen, Lanmei Gao, Xiaojie Chen, Chuan Yan, Rongping Ye, Yueming Li

**Affiliations:** ^1^ Department of Radiology, The First Affiliated Hospital of Fujian Medical University, Fuzhou, Fujian, China; ^2^ School of Medical Imaging, Fujian Medical University, Fuzhou, China; ^3^ Key Laboratory of Radiation Biology (Fujian Medical University), Fujian Province University, Fuzhou, Fujian, China

**Keywords:** hepatocellular carcinoma, glypican-3, radiomics, machine learning, magnetic resonance imaging

## Abstract

**Objectives:** The aim of this study is to investigate the value of multi-phase contrast-enhanced magnetic resonance imaging (CE-MRI) based on the delta radiomics model for identifying glypican-3 (GPC3)-positive hepatocellular carcinoma (HCC).

**Methods:** One hundred and twenty-six patients with pathologically confirmed HCC (training cohort: *n* = 88 and validation cohort: *n* = 38) were retrospectively recruited. Basic information was obtained from medical records. Preoperative multi-phase CE-MRI images were reviewed, and the 3D volumes of interest (VOIs) of the whole tumor were delineated on non-contrast T1-weighted imaging (T1), arterial phase (AP), portal venous phase (PVP), delayed phase (DP), and hepatobiliary phase (HBP). One hundred and seven original radiomics features were extracted from each phase, and delta-radiomics features were calculated. After a two-step feature selection strategy, radiomics models were built using two classification algorithms. A nomogram was constructed by combining the best radiomics model and clinical risk factors.

**Results:** Serum alpha-fetoprotein (AFP) (*p* = 0.013) was significantly related to GPC3-positive HCC. The optimal radiomics model is composed of eight delta-radiomics features with the AUC of 0.805 and 0.857 in the training and validation cohorts, respectively. The nomogram integrated the radiomics score, and AFP performed excellently (training cohort: AUC = 0.844 and validation cohort: AUC = 0.862). The calibration curve showed good agreement between the nomogram-predicted probabilities and GPC3 actual expression in both training and validation cohorts. Decision curve analysis further demonstrates the clinical practicality of the nomogram.

**Conclusion:** Multi-phase CE-MRI based on the delta-radiomics model can non-invasively predict GPC3-positive HCC and can be a useful method for individualized diagnosis and treatment.

## Introduction

Hepatocellular carcinoma (HCC) accounts for 75%–85% of primary liver cancers, which is the sixth most common malignant tumor in humans and the third leading cause of cancer death worldwide (Sung et al., 2021). Despite advances in diagnosis and treatment, the prognosis of HCC patients is still unsatisfied. Hepatectomy and transplantation are considered as the most recommended surgical approaches for HCC treatment (Yang et al., 2019). Unfortunately, the 5-year recurrence rates after surgical resection still reach up to 70% (Llovet et al., 2021).

Glypican-3 (GPC3) is a member of the heparan sulfate proteoglycan family and is overexpressed in most HCC but not in healthy or nonmalignant livers (Capurro et al., 2003). It can help distinguish alpha-fetoprotein (AFP)-negative HCC from benign nodules, suggesting that GPC3 is a more reliable biomarker than AFP in diagnosing HCC (Llovet et al., 2006; Wang et al., 2006). Furthermore, previous studies have shown that GPC3-positive HCC patients have a worse prognosis (Shirakawa et al., 2009; Yorita et al., 2011; Ning et al., 2012; Fu et al., 2013). It is one of the most popular targets in the treatment of HCC in recent years, and there are several clinical traits that reported that GPC3 shows great potential to be an immunotherapeutic target for HCC (Ho and Kim, 2011; Zhu et al., 2013; Du et al., 2021). Accordingly, GPC3 plays a vital role in the diagnosis, treatment, and prognosis of HCC. Identifying the expression of GPC3 as soon as possible is of great importance to the clinical management of HCC.

Currently, accurate detection of GPC3 expression is mainly achieved through postoperative immunohistochemical examination. Although needle biopsy can test GPC3 expression before surgery, it is an invasive method that cannot reflect the heterogeneity of the entire tumor and is susceptible to sampling variation (Zhou et al., 2018). Recently, several studies have found that GPC3 can be released from the cell surface into peripheral blood, indicating the potential of using serum GPC3 levels in HCC diagnosis (Capurro et al., 2003). However, the results vary among studies and need to be further verified (Zhou et al., 2018; Guo et al., 2020). Thus, a preoperative and non-invasive method is needed for predicting the expression of GPC3.

Radiomics, an emerging imaging technology that employs cutting-edge computational tools to extract high-throughput quantitative imaging features and builds predictive models via statistical ways or machine learning to improve diagnosis and prognosis prediction, is attracting increasing attention in cancer research studies (Lambin et al., 2017). Recently, plenty of research studies utilizing the radiomics method demonstrated favorable performance in preoperatively predicting KI67, CK19, microvascular invasion (MVI), and other pathological factors in HCC (Li et al., 2019; Wang et al., 2020; Chong et al., 2021; Zhu et al., 2021). Furthermore, delta radiomics, which integrates time components and radiomics features, provides additional information about the evolution of feature values (Fave et al., 2017; Mokrane et al., 2020). To the best of author’s knowledge, only one study (Gu et al., 2020) has preoperatively predicted GPC3 expression by radiomics features extracted from delayed-phase magnetic resonance imaging (MRI) images. The values of other phases and delta information have not been explored yet.

The present study aims to investigate the performance of the radiomics model based on multi-phase contrast-enhanced magnetic resonance imaging (CE-MRI) and evaluate the effect of delta-radiomics features in predicting GPC3 positive HCC preoperatively.

## Materials and methods

### Patients

**FIGURE 1:**
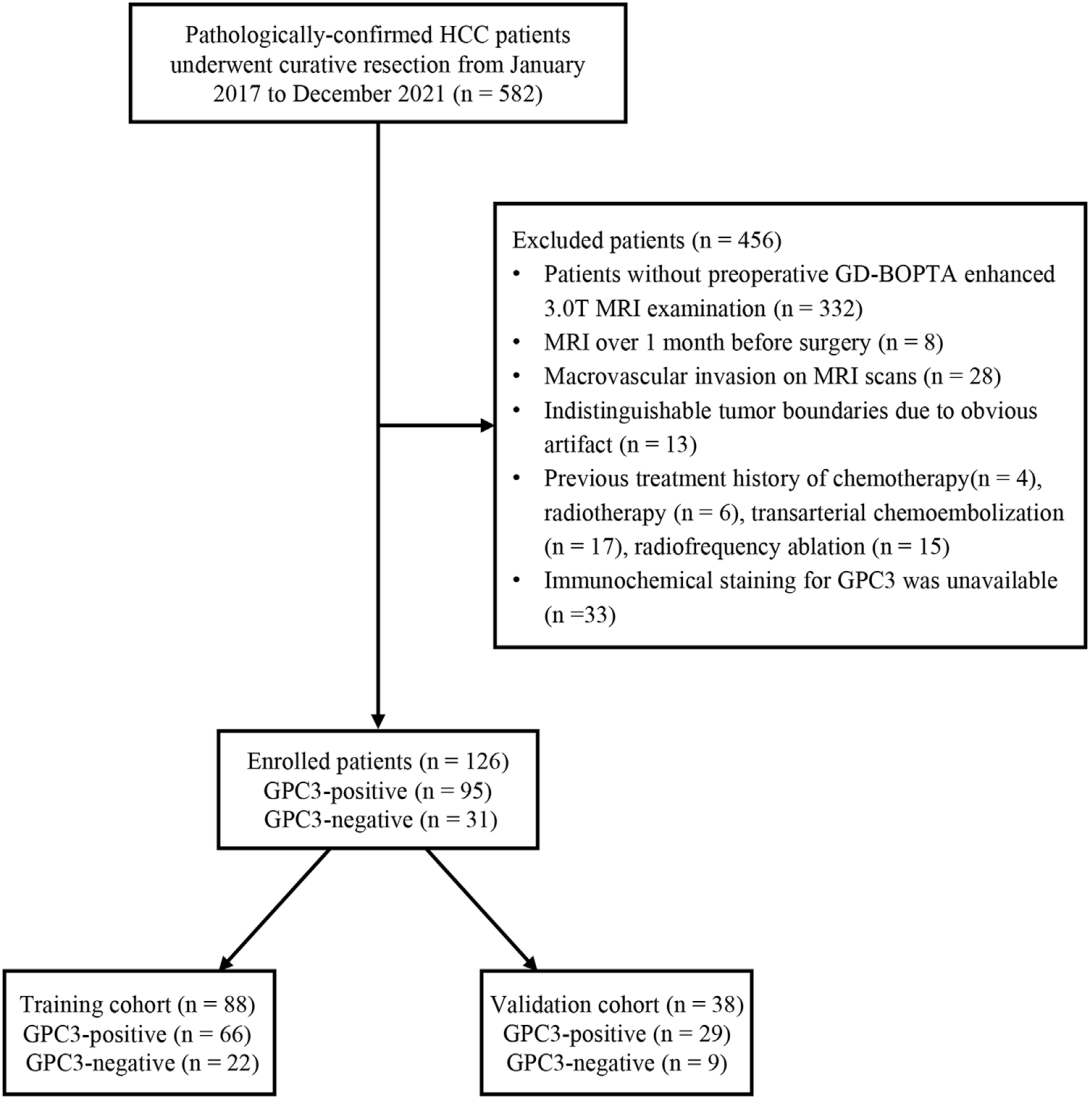
Flow chart of the enrolled patients in our study. HCC, hepatocellular carcinoma; GPC3, glypican-3; GD-BOPTA, gadobenate dimeglumine; MRI, magnetic resonance imaging.

This retrospective study was approved by the institutional review board, and the requirement for informed consent was waived. From January 2017 to December 2021, 582 pathologically confirmed HCC patients who underwent curative resection were consecutively enrolled. The inclusion criterion is as follows: patients with stage Ia, Ib, and IIa hepatocellular carcinoma according to the China liver cancer (CNLC) staging system (Zhou et al., 2020) who underwent surgical resection as first-line treatment. The exclusion criteria are listed as follows: 1) patients who had no preoperative gadobenate dimeglumine (GD-BOPTA)-enhanced 3.0T MRI examination; 2) MRI was performed more than a month before surgery; 3) patients with macrovascular invasion upon preoperative MRI examination; 4) patients with indistinguishable tumor boundaries due to obvious artifacts on MRI images; 5) HCC patients who had previous treatment such as chemotherapy, radiotherapy, transarterial chemoembolization (TACE), and radiofrequency ablation; and 6) immunochemical staining for GPC3 was unavailable. The status of GPC3 expression was recorded according to the pathological report and was categorized into GPC3-positive or GPC3-negative HCC patients. Finally, 126 HCC patients who met the criteria were included and randomly split into the training cohort (*n* = 88) and validation cohort (*n* = 38) at a ratio of 7:3 based on the stratified method (Figure 1).

### Laboratory test and MRI protocol

According to the hospital information system, we collected patients’ basic information, preoperative blood test, and biochemical results, including age, gender, hepatitis B and C immunology, cirrhosis, AFP level, platelet count (PLT), prothrombin time (PT), international normalized ratio (INR), total bilirubin (TBIL), serum albumin (ALB), and alanine aminotransferase/aspartate aminotransferase (ALT/AST).

All patients experienced preoperative liver CE-MRI using the same 3.0T MRI scanner. Image acquisition sequences including transverse T2-weighted imaging (T2WI) with fat suppression, diffusion-weighted imaging (DWI), in-phase and opposed-phase T1-weighted imaging (T1WI), pre-contrast three-dimensional volumetric-interpolated breath-hold T1WI, and T1WI after contrast medium injection (arterial phase, AP, 20–30 s; portal venous phase: PVP, 60–70 s; delayed phase, DP, 2–3 min; hepatobiliary phase, HBP, 90 min). The detailed parameters are explained in the Supplementary Method.

### Histopathological analysis

Histopathological evaluation was available after hepatectomy for HCC across all patients. At the participating hospital, all surgical specimens were routinely fixed in a 10% formaldehyde solution. Two pathologists who were blind to MRI information jointly evaluated the surgical specimens using a standard seven-point sampling method (Liao et al., 2021). A mouse anti-human glypican-3 monoclonal antibody (#MAB-0617 Maixin-Bio, Fujian, China) was used for immunohistochemical labeling of GPC3. GPC3 staining was considered positive when the brown reaction product was present in at least 1 (tumoral) hepatocyte (Libbrecht et al., 2006).

### Radiological assessment

All MR images were reviewed independently by two abdominal radiologists (X.J.C with 3 years of abdominal MRI experience and C.Y with 10 years of abdominal MRI experience) who were aware of the diagnosis of HCC but blinded to the status of GPC3 expression and other clinical data. Divergences between two readers were discussed until a final consensus was achieved. Radiological features in accordance with the Liver Imaging Reporting and Data System (version 2018) (Chernyak et al., 2018) were assessed as follows: (a) tumor margins; (b) tumor capsule; (c) arterial phase hyperenhancement; (d) non-peripheral washout; (e) peritumoral arterial enhancement; (f) tumor hypointensity on HBP; (g) peritumoral hypointensity on HBP; (h) mosaic architecture; (i) intratumoral fat; (j) intratumoral hemorrhage; and (k) intratumoral necrosis.

### Clinical–radiological model

All clinical factors and qualitative radiological features in the training cohort were analyzed first using univariate logistic regression analyses. Those factors with a *p* value less than 0.05 in univariate analyses were entered into multivariate logistic regression analyses to find the independent predictors, and then the clinical–radiological model was built. The predictive capacity of the clinical–radiological model was further evaluated in the validation cohort.

### Radiomics analysis

**FIGURE 2:**
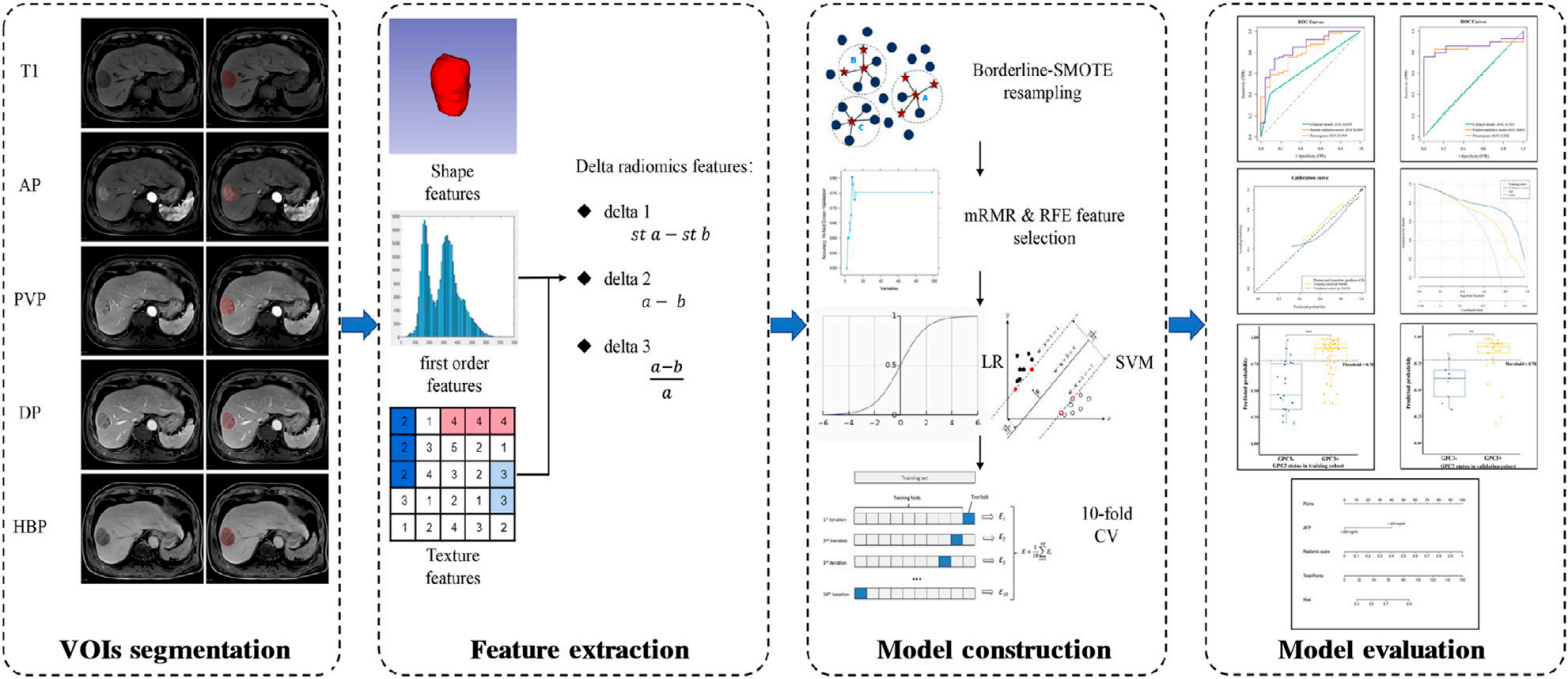
Flow chart of radiomics analysis. T1, non-contrast T1-weighted imaging; AP, arterial phase; PVP, portal venous phase; DP, delayed phase; HBP, hepatobiliary phase; st a, standardized a [a means radiomics features from a single phase (e.g., radiomics features from AP)]; st b, standardized b [b means radiomics features from another single phase (e.g., radiomics features from T1)]; SMOTE, synthetic minority over-sampling technique; mRMR, minimal-redundancy-maximal-relevance; RFE, recursive feature elimination; LR, logistic regression; SVM, support vector machine; CV, cross-validation.

The main flow chart of radiomics analysis is shown in Figure 2. The N4 bias-field correction algorithm was applied to correct the inhomogeneity of MR images. The volumes of interest (VOIs), defined as the whole tumor without peritumoral vessels or bile ducts, were manually delineated by a radiologist (X.J.C) on T1WI, AP, PVP, DP, and HBP images using 3D Slicer software (https://www.slicer.org/). The details of VOIs segmentation are shown in the Supplementary Method. If multiple lesions were found, only the largest one was delineated. After an interval of 2 months, repeated segmentation was performed on 30 randomly selected patients by another radiologist (H.T.D). The two radiologists who perform the segmentation were all blind to the GPC3 status and other clinical information. The segmentation reproducibility was assessed using the Dice similarity coefficient (DSC), and the reproducibility of radiomics features was assessed by the intra-class correlation coefficient (ICC).

Image preprocessing and feature extraction were performed using the home-made python project based on the open-source package PyrRadiomics (version 3.0.1, https://www.radiomics.io/pyradiomics.html). Image normalization and spatial resampling were performed before radiomics-feature extraction in order to normalize the image intensity values and standardize the voxel spacing. We extracted 107 original radiomics features (19 first order features, 13 shape features, and 75 texture features). Detailed descriptions of radiomics features can be found online (https://pyradiomics.readthedocs.io/en/latest/features.html). Additionally, we calculated delta features, which are defined as feature value changes between different phases (Mokrane et al., 2020). Three types of delta features were calculated as follows: 1) standardized subtraction (delta1); 2) direct subtraction (delta2); and 3) relative subtraction (delta3). In each patient, a total of 2,782 features were extracted from 26 phases (sequences), including non-contrast T1WI (T1), AP, PVP, DP, and HBP; _delta1_AP-T1, _delta1_PVP-T1, _delta1_DP-T1, _delta1_HBP-T1, _delta1_PVP-AP, _delta1_DP-PVP, and _delta1_HBP-DP; _delta2_AP-T1, _delta2_PVP-T1, _delta2_DP-T1, _delta2_HBP-T1, _delta2_PVP-AP, _delta2_DP-PVP, and _delta2_HBP-DP; _delta3_AP-T1, _delta3_PVP-T1, _delta3_DP-T1, _delta3_HBP-T1, _delta3_PVP-AP, _delta3_DP-PVP, and _delta3_HBP-DP.

The ratio of GPC3-negative patients to GPC3-positive patients is 1:3.065 in this study, which reveals a data imbalance. Thus, we used Synthetic Minority Over-sampling TEchnique (SMOTE) method (‘BorderlineSMOTE’ packages from scikit-learn) to balance the GPC3-negative group in the training cohort. Subsequently, features in the training cohort were normalized by the z-score, and features in the validation cohort were normalized using the mean and standard deviation values derived from the training cohort. We utilized a two-step feature selection procedure to reduce feature dimension and select robust features. First, the minimal-redundancy-maximal-relevance (mRMR) algorithm (‘pymrmr’ packages in the Python project) was recruited to rank feature importance. Briefly, input features were ranked by maximizing mutual information (MI) to class labels and minimizing MI with other features (Peng et al., 2005). The top-20 features ranked by mRMR were used for further selection by recursive feature elimination (RFE) algorithm with 10-fold cross-validation (‘RFECV’ packages from scikit-learn).

We built preliminary models based on five phases, namely, delta1, delta2, and delta3 features, respectively, using logistic regression (LR) and support vector machine (SVM). Indicators such as area under the receiver operating characteristic (ROC) curve (AUC), accuracy, sensitivity, and specificity were used for model evaluation. Subsequently, features in the preliminary model with the AUC higher than 0.75 in both the training cohort and validation cohort were combined to construct the fusion model. The fusion model with the best discriminative power was selected as the final model, and the prediction probability of the final model was used as radiomics signature.

### Nomogram construction and evaluation

A nomogram was built by integrating the clinical–radiological risk factors and the radiomics signature in the training cohort into multivariable logistic regression and was assessed in the validation cohort. Calibration curves were utilized to analyze the agreement between the predicted and observed GPC3 status. Decision curve analysis was conducted to determine the clinical utility of the nomogram.

### Statistical analyses

Continuous variables with normal distribution were compared by using the Student’s t-test and those with non-normal distribution were compared by using the Mann–Whitney *U*-test. Categorical variables were compared using the chi-squared test or Fisher’s exact test. The DeLong test was used to compare the AUC between different models. Hosmer–Lemeshow test (HL test) was used to evaluate the goodness of fit of the nomogram. All statistical analyses were performed using R software (version 4.1.0; http://www.r-project.org). Two-sided *p* < 0.05 was considered to indicate statistical significance.

## Results

### Basic characteristics and clinical model performance

Comparisons of clinical and radiological characteristics between the training cohort and the validation cohort are summarized in Table 1. No statistical difference was observed between the two groups (*p* = 0.243–1.0), except for gender (*p* = 0.038) and non-peripheral washout (*p* = 0.023).

**TABLE 1:** Clinical characteristics and radiological features in the training and validation cohorts.

Variable	Training cohort (n = 88)			Validation cohort (n = 38)			*p*_*inter*_
	GPC3− (n = 22)	GPC3+ (n = 66)	*p*_*intra*_	GPC3− (n = 9)	GPC3+ (n = 29)	*p*_*intra*_	
Age (years), mean ± SD	62.5 ± 13.92	57.44 ± 12.38	0.139	62.56 ± 6.84	58.59 ± 11.11	0.210	0.705
Gender			1.000			0.237	0.038
Female	3 (13.64)	11 (16.67)		1 (11.11)	0 (0)		
Male	19 (86.36)	55 (83.33)		8 (88.89)	29 (100)		
Hepatic virus infection			1.000			0.650	0.777
Absent	4 (18.18)	11 (16.67)		1 (11.11)	7 (24.14)		
Present (HBV/HCV)	18 (81.82)	55 (83.33)		8 (88.89)	22 (75.86)		
Cirrhosis			0.604			1.000	0.720
Absent	9 (40.91)	21 (31.82)		2 (22.22)	9 (31.03)		
Present	13 (59.09)	45 (68.18)		7 (77.78)	20 (68.97)		
AFP			0.007			1.000	0.35
≤20 ng/mL	15 (68.18)	22 (33.33)		4 (44.44)	11 (37.93)		
20–400 ng/mL	5 (22.73)	17 (25.76)		3 (33.33)	11 (37.93)		
>400 ng/mL	2 (9.09)	27 (40.91)		2 (22.22)	7 (24.14)		
PLT			0.380			0.396	0.728
≤125 × 10^9^/L	3 (13.64)	16 (24.24)		1 (11.11)	9 (31.03)		
>125 × 10^9^/L	19 (86.36)	50 (75.76)		8 (88.89)	20 (68.97)		
PT			0.589			0.115	0.376
≤13 s	17 (77.27)	45 (68.18)		3 (33.33)	20 (68.97)		
>13 s	5 (22.73)	21 (31.82)		6 (66.67)	9 (31.03)		
INR			0.139			0.396	0.622
≤1.0	7 (31.82)	11 (16.67)		1 (11.11)	9 (31.03)		
>1.0	15 (68.18)	55 (83.33)		8 (88.89)	20 (68.97)		
TBIL			1.000			0.456	0.326
≤20.5 μmol/L	17 (77.27)	50 (75.76)		7 (77.78)	18 (62.07)		
>20.5 μmol/L	5 (22.73)	16 (24.24)		2 (22.22)	11 (37.93)		
ALB			0.806			0.703	0.728
≤40 g/L	12 (54.55)	32 (48.48)		5 (55.56)	12 (41.38)		
>40 g/L	10 (45.45)	34 (51.52)		4 (44.44)	17 (58.62)		
ALT/AST, median (Q1 and Q3)	0.98 (0.76, 1.22)	1 (0.75, 1.29)	0.633	1.34 (1.18, 1.4)	1.09 (0.75, 1.21)	0.186	0.468
Maximum tumor length (cm), median (Q1 and Q3)	60.08 (43.23, 78.6)	50 (28.91, 73.9)	0.381	72.47 (58.31, 93.51)	36.77 (30.59, 51.92)	0.003	0.515
Tumor margins			0.281			0.650	0.443
Smooth	9 (40.91)	17 (25.76)		1 (11.11)	7 (24.14)		
Non-smooth	13 (59.09)	49 (74.24)		8 (88.89)	22 (75.86)		
Tumor capsule			0.903			0.481	0.843
Complete	5 (22.73)	19 (28.79)		1 (11.11)	9 (31.03)		
Absent	5 (22.73)	14 (21.21)		2 (22.22)	8 (27.59)		
Incomplete	12 (54.55)	33 (50)		6 (66.67)	12 (41.38)		
APHE			0.894			1.000	0.064
Absent	6 (27.27)	21 (31.82)		1 (11.11)	4 (13.79)		
Present	16 (72.73)	45 (68.18)		8 (88.89)	25 (86.21)		
Non-peripheral washout			0.386			0.650	0.023
Absent	12 (54.55)	27 (40.91)		1 (11.11)	7 (24.14)		
Present	10 (45.45)	39 (59.09)		8 (88.89)	22 (75.86)		
Peritumoral arterial enhancement			1.000			0.148	0.658
Absent	9 (40.91)	25 (37.88)		2 (22.22)	15 (51.72)		
Present	13 (59.09)	41 (62.12)		7 (77.78)	14 (48.28)		
Tumor hypointensity on HBP			0.440			0.554	0.067
Absent	1 (4.55)	1 (1.52)		0 (0)	4 (13.79)		
Present	21 (95.45)	65 (98.48)		9 (100)	25 (86.21)		
Peritumoral hypointensity on HBP			1.000			1.000	0.945
Absent	15 (68.18)	43 (65.15)		6 (66.67)	20 (68.97)		
Present	7 (31.82)	23 (34.85)		3 (33.33)	9 (31.03)		
Mosaic architecture			0.589			0.273	0.376
Absent	5 (22.73)	21 (31.82)		2 (22.22)	13 (44.83)		
Present	17 (77.27)	45 (68.18)		7 (77.78)	16 (55.17)		
Intratumoral fat			0.766			0.075	0.470
Absent	17 (77.27)	53 (80.3)		6 (66.67)	27 (93.1)		
Present	5 (22.73)	13 (19.7)		3 (33.33)	2 (6.9)		
Intratumoral hemorrhage			0.173			0.699	0.560
Absent	9 (40.91)	40 (60.61)		5 (55.56)	19 (65.52)		
Present	13 (59.09)	26 (39.39)		4 (44.44)	10 (34.48)		
Intratumoral necrosis			1.000			0.052	0.728
Absent	13 (59.09)	38 (57.58)		3 (33.33)	21 (72.41)		
Present	9 (40.91)	28 (42.42)		6 (66.67)	8 (27.59)		

Unless indicated otherwise, data are number of patients with percentages in parentheses. *p*_*intra*_ represents the *p* value between GPC3+ and GPC3− groups; *p*_*inter*_ represents the *p* value between the training cohort and the validation cohort. HBV, hepatitis B virus; HCV, hepatitis C virus; AFP, serum alpha-fetoprotein; PLT, platelet count; PT, prothrombin time; INR, international normalized ratio; TBIL, serum total bilirubin; ALB, serum albumin; ALT, alanine aminotransferase; AST, aspartate aminotransferase; APHE, arterial phase hyperenhancement; HBP, hepatobiliary phase.

**FIGURE 3:**
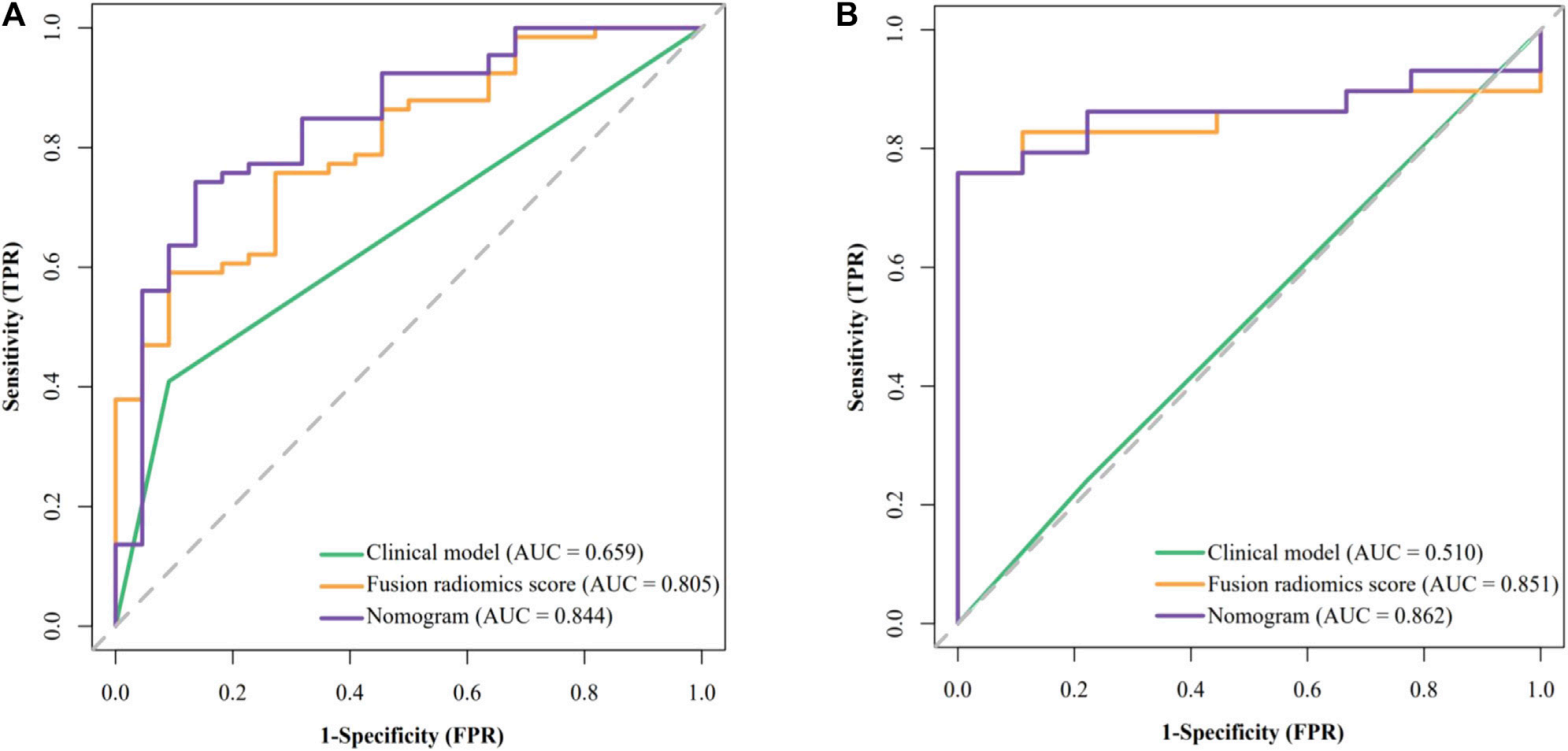
Receiver operating characteristic (ROC) curves of the clinical model, fusion radiomics score, and nomogram. **(A)** Training cohort and **(B)** validation cohort.

The results of univariate analysis (see Supplementary Table S1) showed that only AFP is significantly related to GPC3 expression (OR = 6.923 [95% CI: 1.814–45.64] and *p* = 0.013). None of the radiological features were significantly related to GPC3 (*p* = 0.111–0.901). Thus, AFP was used as the clinical model and had an AUC (95% CI) of 0.659 (0.573–0.745), accuracy of 0.534, sensitivity of 0.409, and specificity of 0.909 in the training cohort (Figure 3A). In validation cohort, the AUC (95% CI), accuracy, sensitivity, and specificity of the clinical model were 0.598 (0.388–0.807), 0.737, 0.862, and 0.333, respectively (Figure 3B).

### Construction and evaluation of radiomics models

First, preliminary models were constructed and evaluated. The performance of each preliminary model in the training cohort and the validation cohort is shown in Supplementary Table S2. Among five single-phase radiomics models, only the one derived from DP has an AUC higher than 0.75 in both the training and the validation cohort. In delta1, no model met the criteria. In delta2 and delta3, models based on _delta2_AP-t1, _delta2_PVP-t1, _delta2_HBP-t1, _delta2_PVP-AP, _delta3_PVP-t1, and _delta3_HBP-t1 showed satisfactory results (AUC higher than 0.75 in both training and validation cohorts) and were used in constructing the fusion models according to permutation and combination.

The performance of all fusion models is presented in Table 2. The logistic regression model based on _delta2_AP-T1, _delta2_HBP-T1, and _delta2_PVP-AP (fusion9) showed the best overall performance, achieving AUCs of 0.862 (95% CI: 0.795–0.912) and 0.851 (95% CI: 0.717–0.959) for the training cohort (“SMOTE” training cohort) and the validation cohort, respectively, which was named as the optimal model in the following section. The selected features and their corresponding coefficients are shown in Table 3. Radiomics score was calculated from the predicted probability of the optimal model. The AUCs of radiomics score were 0.805 in the training cohort and 0.851 in the validation cohort (Figures 3A, B).

**TABLE 2:** Performance of all fusion models.

Models	Fusion phase	Classifier	Training cohort				Validation cohort			
			AUC (95%CI)	Accuracy	Sensitivity	Specificity	AUC (95%CI)	Accuracy	Sensitivity	Specificity
Fusion 1	_delta2_AP-T1 and _delta2_PVP-T1	LR	0.839 [0.767, 0.903]	0.750	0.576	0.939	0.751 [0.558, 0.917]	0.763	0.759	0.778
		SVM	0.813 [0.734, 0.888]	0.758	0.606	0.924	0.789 [0.625, 0.929]	0.711	0.655	0.889
Fusion 2	_delta2_AP-T1 and _delta2_HBP-T1	LR	0.846 [0.770, 0.911]	0.811	0.742	0.894	0.812 [0.639, 0.943]	0.737	0.759	0.667
		SVM	0.804 [0.721, 0.874]	0.758	0.697	0.833	0.774 [0.604, 0.922]	0.711	0.690	0.778
Fusion 3	_delta2_AP-T1 and _delta2_PVP-AP	LR	0.789 [0.707, 0.861]	0.758	0.591	0.939	0.766 [0.622, 0.904]	0.684	0.621	0.889
		SVM	0.760 [0.675, 0.840]	0.727	0.636	0.833	0.762 [0.588, 0.912]	0.632	0.621	0.667
Fusion 4	_delta2_PVP-T1 and _delta2_HBP-T1	LR	0.823 [0.749, 0.889]	0.765	0.667	0.879	0.701 [0.488, 0.897]	0.658	0.621	0.778
		SVM	0.811 [0.739, 0.880]	0.735	0.727	0.758	0.609 [0.368, 0.828]	0.579	0.586	0.556
Fusion 5	_delta2_PVP-T1 and _delta2_PVP-AP	LR	0.754 [0.673, 0.830]	0.720	0.576	0.879	0.766 [0.596, 0.916]	0.711	0.724	0.667
		SVM	0.752 [0.672, 0.830]	0.712	0.773	0.667	0.818 [0.658, 0.958]	0.816	0.862	0.667
Fusion 6	_delta2_HBP-T1 and _delta2_PVP-AP	LR	0.854 [0.789, 0.913]	0.788	0.818	0.773	0.778 [0.593, 0.936]	0.763	0.828	0.556
		SVM	0.858 [0.790, 0.919]	0.811	0.803	0.833	0.808 [0.627, 0.952]	0.763	0.793	0.667
Fusion 7	_delta2_AP-T1 and _delta2_PVP-T1 and _delta2_HBP-T1	LR	0.882 [0.816, 0.931]	0.833	0.742	0.939	0.808 [0.627, 0.954]	0.763	0.724	0.889
		SVM	0.906 [0.846, 0.960]	0.864	0.833	0.909	0.808 [0.581, 0.975]	0.816	0.793	0.889
Fusion 8	_delta2_AP-T1 and _delta2_PVP-T1 and _delta2_PVP-AP	LR	0.804 [0.731, 0.875]	0.742	0.682	0.818	0.625 [0.441, 0.800]	0.605	0.655	0.444
		SVM	0.778 [0.693, 0.866]	0.765	0.803	0.742	0.743 [0.562, 0.904]	0.711	0.759	0.556
Fusion 9	_delta2_AP-T1 and _delta2_HBP-T1 and _delta2_PVP-AP	LR*	0.862 [0.795, 0.912]	0.795	0.758	0.848	0.851 [0.717, 0.959]	0.842	0.828	0.889
		SVM	0.866 [0.796, 0.922]	0.818	0.864	0.788	0.659 [0.470, 0.844]	0.711	0.828	0.333
Fusion 10	_delta2_PVP-T1 and _delta2_HBP-T1 and _delta2_PVP-AP	LR	0.805 [0.731, 0.873]	0.735	0.848	0.636	0.732 [0.535, 0.903]	0.711	0.828	0.333
		SVM	0.830 [0.761, 0.895]	0.780	0.727	0.848	0.774 [0.559, 0.938]	0.763	0.793	0.667
Fusion 11	_delta2_AP-T1 and _delta2_PVP-T1 and _delta2_HBP-T1 and _delta2_PVP-AP	LR	0.850 [0.785, 0.910]	0.780	0.712	0.864	0.720 [0.521, 0.890]	0.658	0.655	0.667
		SVM	0.857 [0.791, 0.918]	0.818	0.788	0.864	0.816 [0.630, 0.954]	0.658	0.621	0.778
Fusion 12	_delta3_PVP-T1 and _delta3_HBP-T1)	LR	0.866 [0.800, 0.922]	0.818	0.788	0.864	0.835 [0.697, 0.954]	0.789	0.828	0.667
		SVM	0.860 [0.789, 0.927]	0.811	0.758	0.879	0.743 [0.567, 0.906]	0.763	0.793	0.667

*Models with the best comprehensive performance were used for the construction of the nomogram.

T1, non-contrast T1-weighted imaging; AP, arterial phase; PVP, portal venous phase; HBP, hepatobiliary phase; LR, logistic regression; SVM, support vector machine; AUC, area under the receiver operating characteristic curve.

**TABLE 3:** Selected features and their corresponding coefficients.

Radiomics feature	Coefficient
_delta2_AP-T1 original_glcm_SumEntropy	−1.741
_delta2_AP-T1 original_glcm_Idmn	0.894
_delta2_AP-T1 original_glcm_Imc2	1.264
_delta2_AP-T1 original_gldm_DependenceNonUniformityNormalized	1.295
_delta2_HBP-T1 original_ngtdm_Busyness	0.652
_delta2_HBP-T1 original_firstorder_Variance	0.805
_delta2_PVP-AP original_ngtdm_Coarseness	1.297
_delta2_PVP-AP original_glszm_SmallAreaLowGrayLevelEmphasis	−0.943

Intercept = 0.198.

T1, non-contrast T1-weighted imaging; AP, arterial phase; PVP, portal venous phase; HBP, hepatobiliary phase.

The mean DSC of VOIs segmentation and the mean ICC of radiomics features in different phases are shown in Supplementary Tables S4–S6. The mean DSC of VOIs on five phases was higher than 0.9, and the mean ICC of radiomics features from different delta phases was higher than 0.8. All of the ICC of radiomics features in the optimal model were higher than 0.8.

### Performance of the nomogram

**FIGURE 4:**
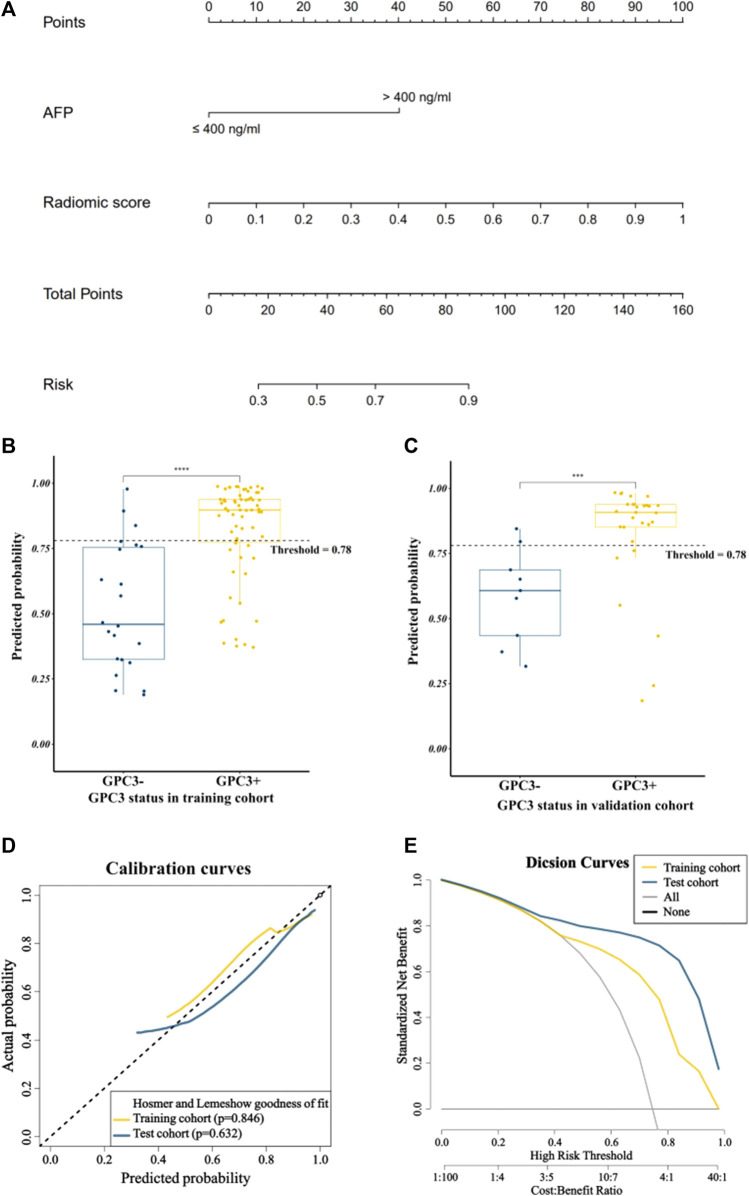
Visualization and evaluation of the comprehensive model. **(A)** Nomogram based on clinical risk factor AFP combined with radiomics score; **(B)** and **(C)** represent box plots showing the distribution of patients’ GPC3-positive probability in the training and validation cohorts; **** for *p* < 0.0001 and *** for *p* < 0.01 by the Mann–Whitney *U* test; **(D)** calibration curves; **(E)** decision curves.

By combining the AFP and radiomics signature of the optimal model through logistic regression, a comprehensive model was built and visualized as a nomogram (Figure 4A). The nomogram yielded an AUC of 0.844 (95% CI: 0.748–0.941) in the training cohort and 0.862 (95% CI: 0.745–0.979) in the validation cohort, respectively, (Figures 3A, B), which were significantly higher than the clinical model (*p* < 0.001 in both cohorts). The accuracy, sensitivity, and specificity of the nomogram in the training cohort and the validation cohort were 0.773, 0.742, and 0.864 and 0.789, 0.793 and, 0.778, respectively. The threshold of the nomogram in the training cohort was 0.78, which was calculated by the Youden index. The box diagram (Figures 2B, C) displayed the distribution of the predicted probability of the nomogram in the GPC3+ group and the GPC3− group and showed statistical difference in both groups (*p* < 0.001 in both cohorts). Calibration curves (Figure 4D) showed good agreement between nomogram-predicted probability and actual GPC3 status in both the training cohort (HL test, *p* = 0.846) and validation cohort (HL test, *p* = 0.632). Decision curve analysis (Figure 4E) demonstrated that the nomogram obtained more clinical net benefits than the strategies of “treat all” and “treat none.”

## Discussion

In this retrospective study, we aim to develop and validate a radiomics-based nomogram to preoperatively predict the status of GPC3 expression in HCC patients. The clinical model, single-phase radiomics models, and delta-radiomics models were built, and delta-radiomics model had higher AUCs than single-phase radiomics models (especially the delta2 radiomics model). The final comprehensive model consisting of _delta2_AP-T1 and _delta2_HBP-T1 and _delta2_PVP-AP radiomics signature and AFP achieved the best overall performance and had more clinical benefits.

Previous studies (Gu et al., 2020; Zhao et al., 2021) have reported that the serum AFP level was significantly associated with GPC3 expression. Our study also has the same result. The potential mechanism may be that the level of GPC3 induction is controlled by alpha-fetoprotein regulator 2 (Afr2) (Morford et al., 2007). During the construction of the clinical model, only the AFP level was significantly related to the GPC3 expression status. Despite the unsatisfactory sensitivity and accuracy, a high specificity of 0.909 was reported in the training cohort of the clinical model based on AFP to predict GPC3 expression. The final comprehensive model, integrated AFP, displayed good performance as well.

Due to the imbalanced proportion of GPC3 expression in our data, we employed the “BorderlineSMOTE” strategy (Wang et al., 2015) to solve the problem. The BorderlineSMOTE algorithm is an improved and scientific oversampling algorithm based on SMOTE, which uses only a few samples on the boundary to synthesize new samples, thereby improving the internal distribution of samples. Moreover, we used two classical and common machine learning algorithms, namely, logistic regression and support vector machine to train the radiomics models. In terms of SVM, we used the linear kernel because only it can output the predicted probability of each sample. Our results showed that LR and SVM performed similarly in model development.

In our study, we extracted radiomics features from three-dimensional VOIs. The entire tumor inherently grows spatially and forms heterogeneously; the VOIs certainly incorporate more texture features and geometrical information rather than the two-dimensional regions of interest (Xu et al., 2019). We not only extracted radiomics features from five different phase images but also calculated delta-radiomics features. Delta radiomics may provide more information about the blood supply and metabolism of tumors. As for delta radiomics, our results showed that in the validation cohort, direct subtraction delta features (AUC ranges: 0.548–0.808) and relative subtraction delta features (AUC ranges: 0.464–0.862) had better performance than standardized delta features (AUC ranges: 0.473–0.724). The best combination of delta features is _delta2_AP-T1 and _delta2_HBP-T1 and _delta2_PVP-AP, and most of them were textured features. Among them, IDMN (inverse difference moment normalized) is a measure of the local homogeneity of an image, and IMC2 quantify the complexity of the texture. These two features from _delta2_AP-T1 may reflect the texture changes from T1WI to the arterial phase and may correspond to the arterial enhancement. Many previous studies have supported that that hepatobiliary phase hypointensity on liver-specific contrast agent-enhanced MRI increases the diagnostic sensitivity for detecting HCC (Cortis et al., 2016; Li et al., 2021). Features from _delta2_HBP-T1 may imply that tumor tissue cannot uptake GD-BOPTA and thus exhibits hypointensity on the hepatobiliary phase, which contrasts to peritumoral hepatic parenchyma.

Gu et al. (2020) used radiomics features based on MRI delayed phase images to predict GPC3 expression in hepatocellular carcinoma, and the AUCs of the final radiomics model are 0.879 and 0.871 in the training and validation cohorts, respectively. In our study, radiomics models from DP had an AUC of 0.772 and 0.755 in the training and validation cohorts, respectively. The difference between the two studies may be due to the bias of diversified sample populations, parameters of scan machines, and different MRI contrast agent, etc. We further analyzed the predictive effect of the optimal delta-radiomics model in our study and found that the model achieved an AUC of 0.957 [0.907–1] in the subgroup population with hepatocellular carcinoma smaller than 5 cm, which was higher than the radiomics model in Gu’s study.

Recently, adoptive cell therapy is emerging in advanced HCC, and many clinical trials investigate CAR T cells targeting GPC3 (Rochigneux et al., 2021). An effective preoperative estimation of GPC3 presence can assist clinicians to choose and customize appropriate therapeutic strategies for patients. Chen et al. (2021) used the IDEAL IQ MRI R2* map to evaluate glypican-3 expression and achieved an AUC of 0.881, sensitivity of 0.859, and specificity 0.842. However, the IDEAL IQ sequence is not a routine sequence in liver MRI examination, and the usage of R2* needs further validation. Our proposed radiomics-based nomogram is an effective and economical tool to preoperatively predict GPC3 expression and is expected to help clinicians make appropriate treatment decision.

Our study has several limitations. First, our study is a single-center retrospective study. The sample size is relatively small, and external validation in other centers is needed. Second, we only focus on muti-phase CE-MRI radiomics features, and multimodal MRI radiomics features such as T2WI and DWI could be explored in the future. Third, our study used Gd-BOPTA CE-MRI, and the hepatobiliary phase was acquired at about 90 min after contrast medium injection. Further validation based on gadoxetate disodium CE-MRI is needed. Fourth, manual segmentation with semi-automatic segmentation tools still takes a lot of time. Registration and deep learning-based auto-segmentation can be used in future studies.

In conclusion, the multi-phase CE-MRI based on delta-radiomics model can non-invasively predict GPC3-positive HCC. Integrated with the serum AFP level, the comprehensive nomogram achieved a satisfactory prediction of GPC3 expression status, which will be beneficial for clinical treatment decision making.

## Figures and Tables

**Table:** 

**AFP**	Alpha-fetoprotein
**ALB**	Albumin
**ALT**	Alanine aminotransferase
**AP**	Arterial phase
**AST**	Aspartate aminotransferase
**AUC**	Area under the curve
**CE-MRI**	Contrast-enhanced magnetic resonance imaging
**DP**	Delayed phase
**DWI**	Diffusion-weighted imaging
**GD-BOPTA**	Dadobenate dimeglumine
**GPC3**	Glypican-3
**HBP**	Hepatobiliary phase
**HCC**	Hepatocellular carcinoma
**ICC**	Intra-class correlation coefficient
**INR**	International normalized ratio
**LR**	Logistic regression
**mRMR**	Minimal-redundancy-maximal-relevance
**MRI**	Magnetic resonance imaging
**MVI**	Microvascular invasion
**OR**	Odds ratio
**PLT**	Platelet count
**PT**	Prothrombin time
**PVP**	Portal venous phase
**RFE**	Recursive feature elimination
**ROC**	Receiver operating characteristic
**SMOTE**	Synthetic minority over-sampling technique
**SVM**	Support vector machine
**T1WI**	T1-weighted imaging
**T2WI**	T2-weighted imaging
**TACE**	Transarterial chemoembolization
**TBIL**	Total bilirubin
**VOIs**	Volumes of interest

## Data Availability

The raw data supporting the conclusion of this article will be made available by the authors, without undue reservation.
