# Supplementary material for: Delta-radiomics models based on multi-phase contrast-enhanced magnetic resonance imaging can preoperatively predict glypican-3-positive hepatocellular carcinoma
Source: Front Physiol. 2023 Aug 3;14:1138239. doi: 10.3389/fphys.2023.1138239 (PMC10435992; doi:10.3389/fphys.2023.1138239)
Supplement: Supplementary file 1 [file DataSheet1.docx]

**Supplementary Material**

[Supplementary Method 2](#_Toc138850811)

[Table S1 5](#_Toc138850812)

[Table S2. 7](#_Toc138850813)

[Table S3. 10](#_Toc138850814)

[Table S4 14](#_Toc138850815)

[Table S5 14](#_Toc138850816)

[Table S6 14](#_Toc138850817)

# Supplementary Method

**Detailed MR scanning parameter**

A 3.0T MR machine (MAGNETOM Verio; Siemens, Healthcare, Erlangen, Germany) with a dedicated phased-array body coil was used for MRI. The standard abdominal MRI protocol included: (1) Axial T2-weighted fat-suppressed turbo-spin-echo: Repetition time (TR)/echo time (TE), 4700/79 msec, slice thickness, 5 mm, slice gap, 1mm, FOV, 21 mm × 38 mm; (2) In-phase and out-of-phase axial T1-weighted imaging (T1WI): TR/TE, 133/2.5 msec (in-phase), 6.2 msec (out-phase), slice thickness, 5 mm, slice gap, 1 mm, FOV, 21 mm × 38 mm; (3) Diffusion-weighted imaging (b = 50, 800 sec/mm^2^) performed with a free-breathing single-shot echo-planar technique, TR/TE, 9965/73 msec, slice thickness, 5 mm, slice gap, 1 mm, FOV, 21 mm × 38 mm. MRI system automatically calculated the corresponding ADC maps; and (4) contrast enhanced MRI, a 3D gradient echo sequence with volumetric interpolated breath-hold examination was performed before and after injection of gadobenate dimeglumine (MultiHance; Bracco), at a dose of 0.2 mL/kg and at a rate of 2 mL/sec followed by a 20 mL saline flush with the following parameters: TR/TE, 3.9/1.4 msec, slice thickness 3 mm, slice gap, 0.6 mm, FOV, 25 mm × 38 mm. Hepatic arterial phase (AP), portal venous phase (PVP), equilibrium phase (EP) and HBP images were obtained at 20–30 sec, 70–80 sec, 180 sec and 90 min after contrast medium injection, respectively.

**Detailed method of VOI segmentation**

We employed an independent segmentation of the volume of interest (VOI) in all five phases. To enhance efficiency and save time, we utilized a semi-automatic segmentation method. We leveraged certain applications in 3D slicer, specifically "Fill between slices" and "Level tracing."

"Fill between slices" assists in filling the gaps between segmented slices by interpolating the missing regions. This eliminates the need for radiologists to manually segment the regions slice by slice. By skipping certain intervals between segmented slices, the software automatically fills in the missing layers. However, it should be noted that the automatically filled regions may not perfectly correspond to the actual tumor boundary. Therefore, radiologists are still required to review and modify the filled slices to ensure the completeness of the VOI. Figure S1 show the process of Fill between slices.

"Level tracing" can define an outline where pixels share the same background value as the current background pixel. Typically, it is used to segment continuous blood vessels by leveraging the high contrast between blood vessels and surrounding tissues. In the case of multi-phase contrast-enhanced MRI images, hepatocellular carcinoma (HCC) exhibits different signal intensities compared to the adjacent liver parenchyma, resulting in distinct pixel values. "Level tracing" can identify these differences and automatically segment the tumor boundary. Similar to the previous method, radiologists need to review and adjust the automatically segmented ROI as necessary.


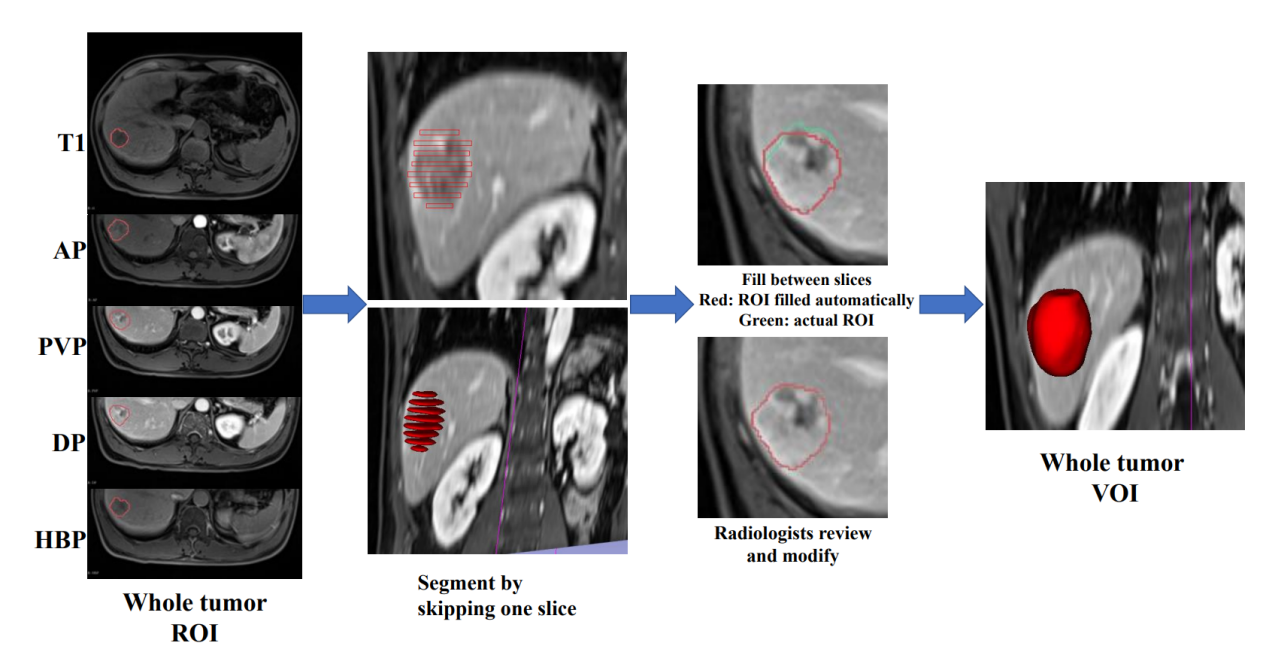


Figure S1. flowchart of VOI segmentation

# Table S1

Univariate analyses of all clinical factors and qualitative radiological features in training cohort.

| Variables | OR (95%CI) | *p* |
| --- | --- | --- |
| Age | 0.968(0.927-1.006) | 0.114 |
| Gender |  |  |
| Female | 1.000 |  |
| Male | 0.789(0.166-2.858) | 0.737 |
| AFP |  |  |
| ≤ 20 ng/ml | 1.000 |  |
| 20-400 ng/ml | 2.318(0.734-8.286) | 0.167 |
| > 400 ng/ml | 9.205(2.275-62.503) | 0.006 |
| Cirrhosis |  |  |
| Absent | 1.000 |  |
| Present | 1.484(0.537-4.002) | 0.437 |
| Hepatic virus infection |  |  |
| Absent | 1.000 |  |
| Present (HBV/HCV) | 1.111(0.281-3.718) | 0.87 |
| PLT |  |  |
| ≤ 125 × 10^9^/L | 1.000 |  |
| > 125 × 10^9^/L | 0.493(0.106-1.695) | 0.302 |
| PT |  |  |
| ≤ 13s | 1.000 |  |
| > 13s | 1.587(0.543-5.343) | 0.421 |
| INR |  |  |
| ≤ 1.0 | 1.000 |  |
| > 1.0 | 2.333(0.748-7.028) | 0.133 |
| TBIL |  |  |
| ≤ 20.5 μmol/L | 1.000 |  |
| > 20.5 μmol/L | 1.088(0.362-3.723) | 0.885 |
| ALB |  |  |
| ≤ 40 g/L | 1.000 |  |
| > 40 g/L | 1.275(0.484-3.415) | 0.623 |
| ALT/AST | 0.963(0.351-2.948) | 0.942 |
| Maximum tumor length | 0.993(0.98-1.007) | 0.313 |
| Tumor margins |  |  |
| Smooth | 1.000 |  |
| Non-smooth | 1.995(0.711-5.499) | 0.181 |
| Tumor capsule |  |  |
| Complete | 1.000 |  |
| Absent | 0.737(0.173-3.124) | 0.673 |
| Incomplete | 0.724(0.205-2.284) | 0.593 |
| APHE |  |  |
| Absent | 1.000 |  |
| Present | 0.804(0.258-2.268) | 0.689 |
| Nonperipheral washout |  |  |
| Absent | 1.000 |  |
| Present | 1.733(0.657-4.667) | 0.267 |
| Peritumoral arterial enhancement |  |  |
| Absent | 1.000 |  |
| Present | 1.135(0.414-3.024) | 0.8 |
| Tumor hypointensity on HBP |  |  |
| Absent | 1.000 |  |
| Present | 3.095(0.119-80.596) | 0.431 |
| Peritumoral hypointensity on HBP |  |  |
| Absent | 1.000 |  |
| Present | 1.146(0.419-3.367) | 0.795 |
| Mosaic architecture |  |  |
| Absent | 1.000 |  |
| Present | 0.63(0.187-1.843) | 0.421 |
| Intratumoral fat |  |  |
| Absent | 1.000 |  |
| Present | 0.834(0.27-2.901) | 0.76 |
| Intratumoral hemorrhage |  |  |
| Absent | 1.000 |  |
| Present | 0.45(0.164-1.19) | 0.111 |
| Intratumoral necrosis |  |  |
| Absent | 1.000 |  |
| Present | 1.064(0.402-2.907) | 0.901 |

# Table S2.

Selected radiomics features in preliminary radiomics models with AUC > 0.75 in both cohorts.

| **Phase/**  **sequence** | **Classifier** | **Selected features** |
| --- | --- | --- |
| DP  (n=6) | LR | DP_original_firstorder_10Percentile  DP_original_glrlm_LongRunLowGrayLevelEmphasis  DP_original_glcm_ClusterShade  DP_original_glcm_Idmn  DP_original_gldm_LowGrayLevelEmphasis  DP_original_glszm_ZoneEntropy |
| DP  (n=6) | SVM | DP_original_firstorder_10Percentile  DP_original_glrlm_LongRunLowGrayLevelEmphasis  DP_original_glcm_ClusterShade  DP_original_glcm_Idmn  DP_original_glszm_ZoneEntropy  DP_original_glrlm_GrayLevelNonUniformity |
| _delta2_AP-T1  (n=8) | LR | AP-T1_original_gldm_SmallDependenceLowGrayLevelEmphasis  AP-T1_original_firstorder_Kurtosis  AP-T1_original_glrlm_LongRunHighGrayLevelEmphasis  AP-T1_original_ngtdm_Strength  AP-T1_original_glcm_SumEntropy  AP-T1_original_glcm_Idmn  AP-T1_original_glcm_Imc2  AP-T1_original_gldm_DependenceNonUniformityNormalized  AP-T1_original_ngtdm_Coarseness |
| _delta2_PVP-T1  (n=6) | LR | PVP-T1_original_firstorder_Energy  PVP-T1_original_firstorder_Kurtosis  PVP-T1_original_glrlm_RunLengthNonUniformity  PVP-T1_original_ngtdm_Strength  PVP-T1_original_glszm_SmallAreaEmphasis  PVP-T1_original_ngtdm_Busyness |
| _delta2_HBP-T1  (n=7) | LR | HBP-T1_original_glrlm_RunLengthNonUniformity  HBP-T1_original_gldm_LargeDependenceLowGrayLevelEmphasis  HBP-T1_original_glszm_LargeAreaHighGrayLevelEmphasis  HBP-T1_original_ngtdm_Coarseness  HBP-T1_original_glszm_SizeZoneNonUniformityNormalized  HBP-T1_original_ngtdm_Busyness  HBP-T1_original_firstorder_Variance |
| _delta2_HBP-T1  (n=7) | SVM | HBP-T1_original_glrlm_RunLengthNonUniformity  HBP-T1_original_glszm_LargeAreaHighGrayLevelEmphasis  HBP-T1_original_ngtdm_Coarseness  HBP-T1_original_glrlm_ShortRunLowGrayLevelEmphasis  HBP-T1_original_glszm_SizeZoneNonUniformityNormalized  HBP-T1_original_ngtdm_Busyness  HBP-T1_original_firstorder_Variance |
| _delta2_PVP-AP  (n=7) | LR | PVP-AP_original_glszm_SmallAreaHighGrayLevelEmphasis  PVP-AP_original_ngtdm_Coarseness  PVP-AP_original_firstorder_Maximum  PVP-AP_original_glcm_Correlation  PVP-AP_original_firstorder_Mean  PVP-AP_original_glszm_GrayLevelNonUniformity  PVP-AP_original_glszm_SmallAreaLowGrayLevelEmphasis |
| _delta2_PVP-AP  (n=8) | SVM | PVP-AP_original_glszm_SmallAreaHighGrayLevelEmphasis  PVP-AP_original_ngtdm_Coarseness  PVP-AP_original_firstorder_Maximum  PVP-AP_original_glcm_Correlation  PVP-AP_original_glszm_LargeAreaLowGrayLevelEmphasis  PVP-AP_original_firstorder_Mean  PVP-AP_original_firstorder_10Percentile  PVP-AP_original_glszm_GrayLevelNonUniformity |
| _delta2_DP-PVP  (n=7) | LR | DP-PVP_original_glszm_SizeZoneNonUniformity  DP-PVP_original_glszm_SmallAreaEmphasis  DP-PVP_original_glrlm_RunLengthNonUniformity  DP-PVP_original_gldm_DependenceVariance  DP-PVP_original_glcm_ClusterProminence  DP-PVP_original_glrlm_ShortRunLowGrayLevelEmphasis  DP-PVP_original_firstorder_InterquartileRange |
| _delta3_PVP-T1  (n=4) | LR | PVP-T1_original_glszm_LargeAreaEmphasis  PVP-T1_original_glszm_SizeZoneNonUniformity  PVP-T1_original_glcm_MCC  PVP-T1_original_glcm_InverseVariance |
| _delta3_PVP-T1  (n=8) | SVM | PVP-T1_original_glszm_LargeAreaEmphasis  PVP-T1_original_glszm_GrayLevelNonUniformity  PVP-T1_original_glszm_ZoneVariance  PVP-T1_original_glcm_ClusterProminenceoriginal_ngtdm_Coarseness  PVP-T1_original_glszm_SizeZoneNonUniformity  PVP-T1_original_glcm_MCC  PVP-T1_original_glcm_InverseVariance |
| _delta3_HBP-T1  (n=8) | LR | HBP-T1_original_glszm_SizeZoneNonUniformity  HBP-T1_original_firstorder_Minimum  HBP-T1_original_gldm_GrayLevelNonUniformity  HBP-T1_original_glcm_InverseVariance  HBP-T1_original_glszm_LowGrayLevelZoneEmphasis  HBP-T1_original_glrlm_RunLengthNonUniformity  HBP-T1_original_glrlm_RunLengthNonUniformityNormalized  HBP-T1_original_glcm_Imc2 |
| _delta3_HBP-T1  (n=8) | SVM | HBP-T1_original_glszm_SizeZoneNonUniformity  HBP-T1_original_ngtdm_Busyness  HBP-T1_original_gldm_GrayLevelNonUniformity  HBP-T1_original_glcm_InverseVariance  HBP-T1_original_glszm_LowGrayLevelZoneEmphasis  HBP-T1_original_glrlm_RunLengthNonUniformity  HBP-T1_original_glrlm_RunLengthNonUniformityNormalized  HBP-T1_original_glcm_Imc2 |

# Table S3.

Selected radiomics features in each fusion models

| **Phase/**  **sequence** | **Classifier** | **Selected features** |
| --- | --- | --- |
| _delta2_AP-T1  &  _delta2_PVP-T1  (n=8) | LR | AP-T1_original_glrlm_LongRunHighGrayLevelEmphasis  AP-T1_original_glcm_SumEntropy  AP-T1_original_glcm_Idmn  AP-T1_original_glcm_Imc2  AP-T1_original_gldm_DependenceNonUniformityNormalized  AP-T1_original_ngtdm_Coarseness  PVP-T1_original_firstorder_Energy  PVP-T1_original_glrlm_RunLengthNonUniformity |
| _delta2_AP-T1  &  _delta2_PVP-T1  (n=7) | SVM | AP-T1_original_glcm_Imc2  AP-T1_original_glcm_SumEntropy  AP-T1_original_gldm_DependenceNonUniformityNormalized  AP-T1_original_ngtdm_Coarseness  PVP-T1_original_firstorder_Kurtosis  PVP-T1_original_glrlm_RunLengthNonUniformity  PVP-T1_original_ngtdm_Strength |
| _delta2_AP-T1  &  _delta2_HBP-T1  (n=8) | LR | AP-T1_original_glcm_Idmn  AP-T1_original_ngtdm_Coarseness  HBP-T1_original_glrlm_RunLengthNonUniformity  HBP-T1_original_glszm_LargeAreaHighGrayLevelEmphasis  HBP-T1_original_ngtdm_Coarseness  HBP-T1_original_glszm_SizeZoneNonUniformityNormalized  HBP-T1_original_ngtdm_Busyness  HBP-T1_original_firstorder_Variance |
| _delta2_AP-T1  &  _delta2_HBP-T1  (n=6) | SVM | AP-T1_original_glcm_Imc1  AP-T1_original_glcm_SumEntropy  AP-T1_original_gldm_DependenceNonUniformityNormalized  AP-T1_original_ngtdm_Coarseness  HBP-T1_original_glrlm_RunLengthNonUniformity  HBP-T1_original_ngtdm_Coarseness |
| _delta2_AP-T1  &  _delta2_PVP-AP  (n=7) | LR | AP-T1_original_ngtdm_Strength  AP-T1_original_glcm_SumEntropy  AP-T1_original_glcm_Imc2  AP-T1_original_gldm_DependenceNonUniformityNormalized  AP-T1_original_ngtdm_Coarseness  PVP-AP_original_ngtdm_Coarseness  PVP-AP_original_glszm_SmallAreaLowGrayLevelEmphasis |
| _delta2_AP-T1  &  _delta2_PVP-AP  (n=7) | SVM | AP-T1_original_glcm_Imc1  AP-T1_original_glcm_SumEntropy  AP-T1_original_gldm_DependenceNonUniformityNormalized  AP-T1_original_ngtdm_Coarseness PVP-AP_original_ngtdm_Coarseness  PVP-AP_original_firstorder_Maximum  PVP-AP_original_firstorder_Mean |
| _delta2_PVP-T1  &  _delta2_HBP-T1  (n=8) | LR | PVP-T1_original_firstorder_Energy  PVP-T1_original_glrlm_RunLengthNonUniformity  HBP-T1_original_glrlm_RunLengthNonUniformity  HBP-T1_original_glszm_LargeAreaHighGrayLevelEmphasis  HBP-T1_original_ngtdm_Coarseness  HBP-T1_original_glszm_SizeZoneNonUniformityNormalized  HBP-T1_original_ngtdm_Busyness  HBP-T1_original_firstorder_Variance |
| _delta2_PVP-T1  &  _delta2_HBP-T1  (n=8) | SVM | PVP-T1_original_glcm_Idmn  PVP-T1_original_firstorder_Energy  PVP-T1_original_glrlm_RunLengthNonUniformity  HBP-T1_original_glrlm_RunLengthNonUniformity  HBP-T1_original_glszm_LargeAreaHighGrayLevelEmphasis  HBP-T1_original_ngtdm_Coarseness  HBP-T1_original_ngtdm_Busyness  HBP-T1_original_firstorder_Variance |
| _delta2_PVP-T1  &  _delta2_PVP-AP  (n=5) | LR | PVP-T1_original_glrlm_RunLengthNonUniformity  PVP-T1_original_glszm_SmallAreaEmphasis  PVP-AP_original_ngtdm_Coarseness  PVP-AP_original_glcm_Correlation  PVP-AP_original_glszm_SmallAreaLowGrayLevelEmphasis |
| _delta2_PVP-T1  &  _delta2_PVP-AP  (n=5) | SVM | PVP-T1_original_glrlm_RunLengthNonUniformity  PVP-T1_original_glszm_HighGrayLevelZoneEmphasis  PVP-AP_original_ngtdm_Coarseness  PVP-AP_original_firstorder_Maximum  PVP-AP_original_firstorder_Mean |
| _delta2_HBP-T1  &  _delta2_PVP-AP  (n=8) | LR | HBP-T1_original_glszm_LargeAreaHighGrayLevelEmphasis  HBP-T1_original_ngtdm_Coarseness  HBP-T1_original_glszm_SizeZoneNonUniformityNormalized  HBP-T1_original_ngtdm_Busyness  HBP-T1_original_firstorder_Variance  PVP-AP_original_ngtdm_Coarseness  PVP-AP_original_glcm_Correlation  PVP-AP_original_glszm_SmallAreaLowGrayLevelEmphasis |
| _delta2_HBP-T1  &  _delta2_PVP-AP  (n=8) | SVM | HBP-T1_original_glszm_LargeAreaHighGrayLevelEmphasis  HBP-T1_original_ngtdm_Coarseness  HBP-T1_original_glszm_SizeZoneNonUniformityNormalized  HBP-T1_original_ngtdm_Busyness  HBP-T1_original_firstorder_Variance  PVP-AP_original_ngtdm_Coarseness  PVP-AP_original_glcm_Correlation  PVP-AP_original_firstorder_Mean |
| _delta2_AP-T1  &  _delta2_PVP-T1  &  _delta2_HBP-T1  (n=8) | LR | AP-T1_original_glrlm_LongRunHighGrayLevelEmphasis  AP-T1_original_gldm_DependenceNonUniformityNormalized  AP-T1_original_ngtdm_Coarseness  PVP-T1_original_firstorder_Energy  PVP-T1_original_glrlm_RunLengthNonUniformity  HBP-T1_original_glrlm_RunLengthNonUniformity  HBP-T1_original_ngtdm_Coarseness  HBP-T1_original_ngtdm_Busyness |
| _delta2_AP-T1  &  _delta2_PVP-T1  &  _delta2_HBP-T1  (n=8) | SVM | AP-T1_original_glcm_Imc1  AP-T1_original_ngtdm_Coarseness  PVP-T1_original_glcm_Idmn  PVP-T1_original_glrlm_RunLengthNonUniformity  HBP-T1_original_glrlm_RunLengthNonUniformity  HBP-T1_original_ngtdm_Coarseness  HBP-T1_original_glszm_SizeZoneNonUniformityNormalized  HBP-T1_original_ngtdm_Busyness |
| _delta2_AP-T1  &  _delta2_PVP-T1  &  _delta2_PVP-AP  (n=5) | LR | AP-T1_original_glrlm_LongRunHighGrayLevelEmphasis  AP-T1_original_ngtdm_Strength  PVP-T1_original_glrlm_RunLengthNonUniformity  PVP-AP_original_ngtdm_Coarseness  PVP-AP_original_glszm_SmallAreaLowGrayLevelEmphasis |
| _delta2_AP-T1  &  _delta2_PVP-T1  &  _delta2_PVP-AP  (n=7) | SVM | AP-T1_original_glcm_Imc1  AP-T1_original_glcm_SumEntropy  AP-T1_original_gldm_DependenceNonUniformityNormalized  AP-T1_original_ngtdm_Coarseness  PVP-T1_original_firstorder_Kurtosis  PVP-T1_original_ngtdm_Strength  PVP-AP_original_glszm_GrayLevelNonUniformity |
| _delta2_AP-T1  &  _delta2_HBP-T1  &  _delta2_PVP-AP  (n=8) | LR | AP-T1_original_glcm_SumEntropy  AP-T1_original_glcm_Idmn  AP-T1_original_glcm_Imc2  AP-T1_original_gldm_DependenceNonUniformityNormalized  HBP-T1_original_ngtdm_Busyness  HBP-T1_original_firstorder_Variance  PVP-AP_original_ngtdm_Coarseness  PVP-AP_original_glszm_SmallAreaLowGrayLevelEmphasis |
| _delta2_AP-T1  &  _delta2_HBP-T1  &  _delta2_PVP-AP  (n=8) | SVM | AP-T1_original_gldm_DependenceNonUniformityNormalized  AP-T1_original_ngtdm_Coarseness  HBP-T1_original_ngtdm_Coarseness  HBP-T1_original_glszm_SizeZoneNonUniformityNormalized  HBP-T1_original_firstorder_Variance  PVP-AP_original_ngtdm_Coarseness  PVP-AP_original_glcm_Correlation  PVP-AP_original_firstorder_Mean |
| _delta2_PVP-T1  &  _delta2_HBP-T1  &  _delta2_PVP-AP  (n=7) | LR | PVP-T1_original_glrlm_RunLengthNonUniformity  HBP-T1_original_glszm_LargeAreaHighGrayLevelEmphasis  HBP-T1_original_ngtdm_Coarseness  HBP-T1_original_ngtdm_Busyness  HBP-T1_original_firstorder_Variance  PVP-AP_original_ngtdm_Coarseness  PVP-AP_original_glszm_SmallAreaLowGrayLevelEmphasis |
| _delta2_PVP-T1  &  _delta2_HBP-T1  &  _delta2_PVP-AP  (n=6) | SVM | PVP-T1_original_glcm_Idmn  HBP-T1_original_glszm_LargeAreaHighGrayLevelEmphasis  HBP-T1_original_ngtdm_Coarseness  HBP-T1_original_ngtdm_Busyness  HBP-T1_original_firstorder_Variance  PVP-AP_original_ngtdm_Coarseness |
| _delta2_AP-T1  &  _delta2_PVP-T1  &  _delta2_HBP-T1  &  _delta2_PVP-AP  (n=8) | LR | AP-T1_original_glrlm_LongRunHighGrayLevelEmphasis  AP-T1_original_ngtdm_Strength  AP-T1_original_ngtdm_Coarseness  PVP-T1_original_glrlm_RunLengthNonUniformity  HBP-T1_original_ngtdm_Coarseness  HBP-t1_original_ngtdm_Busyness  PVP-AP_original_ngtdm_Coarseness  PVP-AP_original_glszm_SmallAreaLowGrayLevelEmphasis |
| _delta2_AP-T1  &  _delta2_PVP-T1  &  _delta2_HBP-T1  &  _delta2_PVP-AP  (n=8) | SVM | AP-T1_original_gldm_SmallDependenceLowGrayLevelEmphasis  AP-T1_original_ngtdm_Coarseness  PVP-T1_original_glcm_Idmn  PVP-T1_original_firstorder_Energy  PVP-T1_original_glszm_HighGrayLevelZoneEmphasis  HBP-T1_original_glrlm_RunLengthNonUniformity  HBP-T1_original_glszm_LargeAreaHighGrayLevelEmphasis  PVP-AP_original_ngtdm_Coarseness |
| _delta3_PVP-T1  &  _delta3_HBP-T1  (n=8) | LR | PVP-T1_original_glszm_LargeAreaEmphasis  PVP-T1_original_glszm_SizeZoneNonUniformity  PVP-T1_original_glcm_InverseVariance  HBP-T1_original_glszm_SizeZoneNonUniformity  HBP-T1_original_firstorder_Minimum  HBP-T1_original_gldm_GrayLevelNonUniformity  HBP-T1_original_glcm_InverseVariance  HBP-T1_original_glcm_Imc2 |
| _delta3_PVP-T1  &  _delta3_HBP-T1  (n=8) | SVM | PVP-T1_original_glszm_GrayLevelNonUniformity  PVP-T1_original_glszm_ZoneVariance  PVP-T1_original_glcm_InverseVariance  HBP-T1_original_glszm_SizeZoneNonUniformity  HBP-T1_original_gldm_GrayLevelNonUniformity  HBP-T1_original_glcm_InverseVariance  HBP-T1_original_glrlm_RunLengthNonUniformityNormalized  HBP-T1_original_glcm_Imc2 |

# Table S4

Average Dice value of single phase VOI and average ICC value of single phase radiomics features

|  | T1 | AP | PVP | DP | HBP |
| --- | --- | --- | --- | --- | --- |
| DSC | 0.948 | 0.964 | 0.951 | 0.957 | 0.948 |
| ICC | 0.954 | 0.988 | 0.963 | 0.985 | 0.965 |

T1, pre-contrast T1 weighted imaging; AP, arterial phase; PVP, portal venous phase; DP, delayed phase; HBP, hepatobiliary phase; DSC, Dice similarity coefficient; ICC, intraclass correlation efficient.

# Table S5

Average ICC value of delta radiomics features

|  | AP-T1 | PVP-T1 | DP-T1 | HBP-T1 | PVP-AP | DP-PVP | HBP-DP |
| --- | --- | --- | --- | --- | --- | --- | --- |
| delta1 | 0.945 | 0.907 | 0.947 | 0.885 | 0.93 | 0.936 | 0.976 |
| delta2 | 0.93 | 0.882 | 0.933 | 0.892 | 0.934 | 0.901 | 0.968 |
| delta3 | 0.932 | 0.816 | 0.902 | 0.907 | 0.82 | 0.901 | 0.968 |

T1, pre-contrast T1 weighted imaging; AP, arterial phase; PVP, portal venous phase; DP, delayed phase; HBP, hepatobiliary phase.

# Table S6

ICC of radiomics features in the optimal radiomics model

| Radiomics feature | ICC |
| --- | --- |
| _delta2_AP-T1 original_glcm_SumEntropy | 0.979 |
| _delta2_AP-T1 original_glcm_Idmn | 0.929 |
| _delta2_AP-T1 original_glcm_Imc2 | 0.919 |
| _delta2_AP-T1 original_gldm_DependenceNonUniformityNormalized | 0.998 |
| _delta2_HBP-T1 original_ngtdm_Busyness | 0.872 |
| _delta2_HBP-T1 original_firstorder_Variance | 0.858 |
| _delta2_PVP-AP original_ngtdm_Coarseness | 0.929 |
| _delta2_PVP-AP original_glszm_SmallAreaLowGrayLevelEmphasis | 0.868 |

T1, pre-contrast T1 weighted imaging; AP, arterial phase; PVP, portal venous phase; HBP, hepatobiliary phase; ICC, intraclass correlation efficient.
